# Association between Visceral Adipose Tissue Metabolism and Alzheimer’s Disease Pathology

**DOI:** 10.3390/metabo12030258

**Published:** 2022-03-17

**Authors:** Shin Kim, Hyon-Ah Yi, Kyoung Sook Won, Ji Soo Lee, Hae Won Kim

**Affiliations:** 1 Department of Immunology, School of Medicine, Keimyung University, Daegu 42601, Korea; god98005@gmail.com; 2 Department of Neurology, Dongsan Hospital, Keimyung University, Daegu 42601, Korea; geschwind@dsmc.or.kr; 3 Department of Nuclear Medicine, Dongsan Hospital, Keimyung University, Daegu 42601, Korea; won@dsmc.or.kr (K.S.W.); jisu81kr@naver.com (J.S.L.)

**Keywords:** adipose tissue, Alzheimer’s disease, amyloid-β, glucose metabolism

## Abstract

The visceral adipose tissue (VAT) has been recognized as an endocrine organ, and VAT dysfunction could be a risk factor for Alzheimer’s disease (AD). We aimed to evaluate the association of VAT metabolism with AD pathology. This cross-sectional study included 54 older subjects with cognitive impairment who underwent 2-deoxy-2-[fluorine-18]-fluoro-D-glucose (^18^F-FDG) torso positron emission tomography (PET) and ^18^F-florbetaben brain PET. ^18^F-FDG uptake in VAT on ^18^F-FDG PET images was used as a marker of VAT metabolism, and subjects were classified into high and low VAT metabolism groups. A voxel-based analysis revealed that the high VAT metabolism group exhibited a significantly higher cerebral amyloid-β (Aβ) burden than the low VAT metabolism group. In the volume-of-interest analysis, multiple linear regression analyses with adjustment for age, sex, and white matter hyperintensity volume revealed that ^18^F-FDG uptake in VAT was significantly associated with the cerebral Aβ burden (*β* = 0.359, *p* = 0.007). In conclusion, VAT metabolism was associated with AD pathology in older subjects. Our findings suggest that VAT dysfunction could contribute to AD development.

## 1. Introduction

Alzheimer’s disease (AD) is a progressive, neurodegenerative disorder characterized by the presence of intracellular neurofibrillary tangles and extracellular amyloid-β (Aβ) plaques in the brain [1]. Although the accumulation of Aβ plaques is believed to be one of the factors driving AD pathogenesis, clear pathophysiology of AD delineating the contributions of each pathological protein has not been confirmed [2].

The visceral adipose tissue (VAT) has been recognized as an endocrine organ, and VAT dysfunction could be a risk factor for AD [3]. Epidemiological studies revealed that high adiposity is correlated with an increased risk of developing dementia, including AD [3]. In addition, significant relationships between adipose-derived molecules, such as leptin and adiponectin, and progression of AD have been reported [4,5]. Moreover, it was recently reported that the proinflammatory cytokine derived from VAT plays an important role in the pathogenesis of AD [6]. However, the mechanism by which VAT dysfunction affects the development and progression of AD remains unclear.

Positron emission tomography (PET) with 2-deoxy-2-[fluorine-18]fluoro-D-glucose (^18^F-FDG) can be used noninvasively to evaluate the metabolic activity in the adipose tissue and serve as a surrogate marker of VAT dysfunction [7,8]. Previous studies have demonstrated that VAT metabolism, measured by ^18^F-FDG PET, is related to several diseases, such as metabolic syndrome and cardiovascular disease [9,10]; however, no studies have shown the relationship between VAT metabolism and AD.

Clarifying the role of VAT dysfunction in AD development could provide evidence for developing treatments for preventing or slowing AD progression. Therefore, this study aimed to evaluate the association of VAT metabolism, measured by ^18^F-FDG PET, with AD pathology in elderly subjects.

## 2. Results

### 2.1. Population Characteristics

**Figure 1:**
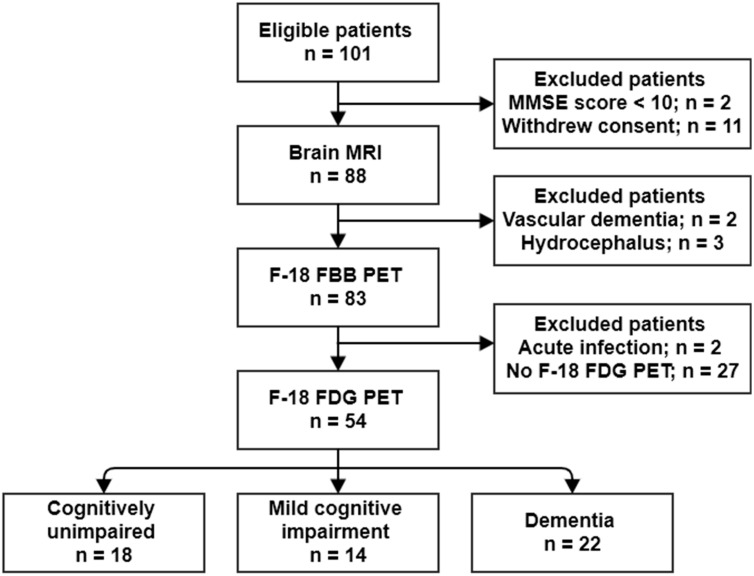
Flow diagram of the study population.

A total of 54 subjects were included in this study (age: 66.4 ± 8.4 years; female, 34 (63.0%)); of whom 18, 14, and 22 were clinically diagnosed as cognitively unimpaired (CU), with mild cognitive impairment (MCI), and with dementia, respectively. A flowchart of the study population is shown in Figure 1. The Mini-Mental State Examination (MMSE) score in the overall cohort was 24.6 ± 5.3. The MMSE score in the dementia group was significantly lower than that in the CU and MCI groups (20.3 ± 5.3 vs. 28.9 ± 1.2, *p* < 0.001; and 20.3 ± 5.3 vs. 25.9 ± 2.9, *p* < 0.001, respectively). The cerebral Aβ burden was quantitatively estimated using volume-of-interest analysis (VOI) on ^18^F-florbetaben (^18^F-FBB) PET images. The regional and composite standardized ^18^F-FBB uptake value ratios (SUVR_FBB_) were calculated. In the overall cohort, 26 subjects (48.1%) were cerebral Aβ-positive on ^18^F-FBB PET images. The rate of patients who were cerebral Aβ-positive in the dementia group (63.6%) was higher than that in the CU (27.8%) and MCI (50.0%) groups; however, it was not significant (*p* = 0.077). Table 1 shows the characteristics of the included patients.

To determine the degree of VAT metabolism, ^18^F-FDG uptake in VAT was measured on torso ^18^F-FDG PET/CT images. The maximum standardized uptake value (SUV_max_) and mean SUV (SUV_mean_) were calculated. The VAT metabolism status was divided by the mean value of VAT SUV_max_ measured in healthy controls in a previous study [11]. A total of 31 subjects (57.4%) were classified into the low VAT metabolism group, and 23 subjects (42.6%) into the high VAT metabolism group. There was no significant difference in clinical variables between the low and high VAT metabolism groups (Table 2).

The composite SUVR_FBB_ was positively correlated with the white matter hyperintensity (WMH) volume (*r* = 0.322, *p* = 0.018), and negatively correlated with the MMSE and Korean version of the Boston Naming Test (K-BNT) scores (*r* = −0.408, *p* < 0.001; and *r* = −0.273, *p* = 0.015; respectively). There was no significant association of the composite SUVR_FBB_ with other variables, including age, sex, body mass index (BMI, kg/m^2^), educational level, diabetes, hypertension, history of cardiovascular disease, and hyperlipidemia. The VAT SUV_max_ and VAT SUV_mean_ were significantly associated with the K-BNT score (*r* = −0.297, *p* = 0.034; and *r* = −0.336, *p* = 0.016; respectively), but not with the MMSE score (*r* = −0.202, *p* = 0.143; and *r* = −0.228, *p* = 0.098; respectively). There was no significant association of the VAT SUV_max_ and VAT SUV_mean_ with other variables, including age, sex, educational level, diabetes, hypertension, history of cardiovascular disease, and hyperlipidemia.

### 2.2. Association of VAT Metabolism with AD Pathology

**Figure 2:**
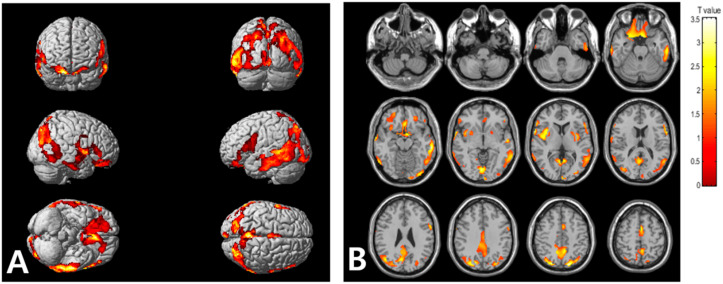
Voxel-based comparison of cerebral Aβ burden between the high and low visceral adipose tissue (VAT) metabolism groups. The statistical parameter mapping t-maps were superimposed on the volume-rendered magnetic resonance imaging (MRI) (**A**) and T1-weighted template in the axial plane (**B**) for the high VAT metabolism group > low VAT metabolism group (*p* < 0.005, uncorrected at voxel-level, cluster size > 100 voxels).

Statistical parametric mapping (SPM) analysis revealed that the high VAT metabolism group, compared with low VAT metabolism group, exhibited significantly high cerebral Aβ burden in the frontal, parietal, temporal, and occipital cortices, as well as in the insula (Figure 2). Table 3 summarizes the brain regions that showed increased cerebral Aβ burden in the high VAT metabolism group, compared with that in the low VAT metabolism group.

In the VOI analysis, the composite SUVR_FBB_ was significantly higher in the high VAT metabolism group than in the low VAT metabolism group (1.60 ± 0.32 vs. 1.34 ± 0.16, *p* < 0.001). The regional SUVR_FBB_ in the bilateral frontal, temporal, and parietal cortices, as well as in the cingulate cortex, were significantly higher in the high VAT metabolism group than in the low VAT metabolism group (Supplementary Table S1). Additionally, the cerebral Aβ-positive group had a significantly higher VAT SUV_max_ and VAT SUV_mean_ than the cerebral Aβ-negative group (0.79 ± 0.14 vs. 0.64 ± 0.15, *p* = 0.001; 0.49 ± 0.10 vs. 0.39 ± 0.10, *p* < 0.001, respectively).

**Figure 3:**
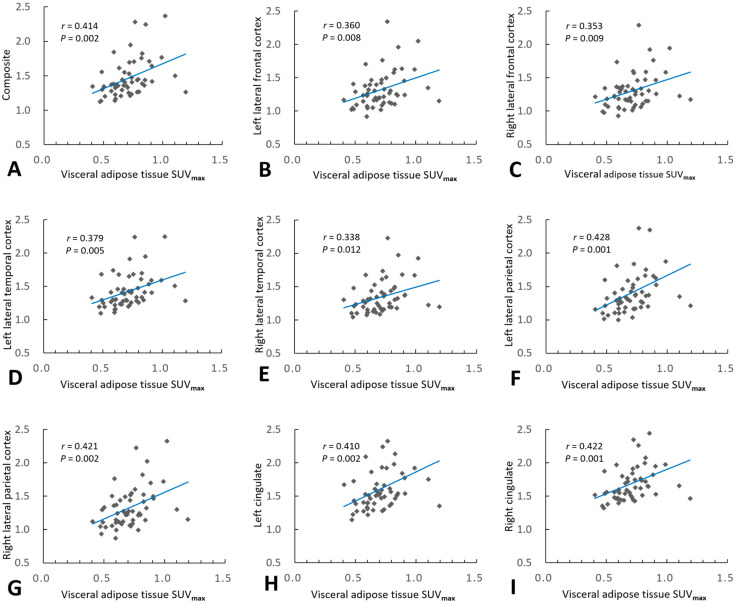
Association of visceral adipose tissue (VAT) metabolism with cerebral amyloid burden. Pearson’s correlation analysis revealed that VAT maximum standardized uptake volume (SUV_max_) correlated positively with composite regional standardized ^18^F-FBB uptake value ratios (SUVR_FBB_) (**A**), and all regional SUVR_FBB_ values in the bilateral lateral frontal (**B**,**C**), lateral temporal (**D**,**E**), and lateral parietal (**F**,**G**) cortices, as well as the bilateral cingulate cortices (**H**,**I**).

In the overall cohort, Pearson’s correlation analyses showed that the VAT SUV_max_ and VAT SUV_mean_ correlated positively with the composite SUVR_FBB_ (*r* = 0.414, *p* = 0.002 and *r* = 0.367, *p* = 0.006; respectively), and the regional SUVR_FBB_ for the lateral frontal, lateral temporal, and lateral parietal cortices, as well as the cingulate cortex (Figure 3). Multiple linear regression analyses, adjusted for age, sex, and WMH volume, revealed that the VAT SUV_max_ was significantly associated with composite SUVR_FBB_ (*β* = 0.359, *p* = 0.007) and all regional SUVR_FBB_ values (Table 4). Additionally, multiple linear regression analyses, adjusted for age, sex, and WMH volume, revealed that the VAT SUV_mean_ was significantly associated with composite SUVR_FBB_ (*β* = 0.295, *p* = 0.032) and SUVR_FBB_ in all regions except the right lateral temporal cortex (Table 5).

In subjects with dementia, Pearson’s correlation analyses showed that the VAT SUV_max_ correlated positively with the composite SUVR_FBB_ (*r* = 0.533, *p* = 0.011), and the SUVR_FBB_ for the lateral frontal, lateral temporal, and lateral parietal cortices, as well as the cingulate cortex. The VAT SUV_mean_ were significantly correlated positively with the SUVR_FBB_ for the lateral frontal cortex, but not with the composite SUVR_FBB_ (*r* = 0.417, *p* = 0.054) or any other regional SUVR_FBB_ in subjects with dementia. Multiple linear regression analyses, adjusted for age, sex, and WMH volume, revealed that VAT SUV_max_ was significantly associated with composite SUVR_FBB_ (*β* = 0.533, *p* = 0.011) and all regional SUVR_FBB_ values in subjects with dementia (Supplementary Table S2). Multiple linear regression analyses, adjusted for age, sex, and WMH volume, revealed that the VAT SUV_mean_ was significantly associated with SUVR_FBB_ for the lateral parietal and cingulate cortices, but not with the composite SUVR_FBB_ or any other regional SUVR_FBB_ in subjects with dementia (Supplementary Table S3). There was no significant correlation of the VAT SUV_max_ and VAT SUV_mean_ with composite SUVR_FBB_ and any of the regional SUVR_FBB_ values in CU subjects or subjects with MCI (Supplementary Tables S4–S7).

## 3. Discussion

VAT, which produces a wide array of bioactive peptides, has been recognized as an endocrine organ [11]. An increase in VAT mass promotes abnormal secretion of adipose-derived inflammatory cytokines or a wide array of bioactive peptides, causing VAT dysfunction [5] that could affect the brain [12]. To the best of our knowledge, no previous study has evaluated the association between VAT metabolism, as measured by ^18^F-FDG PET/CT, and AD pathology. The present study demonstrated that VAT metabolism correlated positively with cerebral Aβ burden. These findings provide strong evidence that VAT dysfunction is related to AD development.

In the present study, VAT metabolism, measured by ^18^F-FDG PET, was used as a surrogate marker of VAT dysfunction. In accordance with the present study, several previous studies reported that VAT metabolism, as measured by ^18^F-FDG PET, is related to several diseases, and that ^18^F-FDG uptake in VAT is an excellent measure of VAT dysfunction. A prospective ^18^F-FDG PET study demonstrated that ^18^F-FDG in the neck adipose tissue was highly predictive of cardiovascular risk in 173 patients [10]. Another ^18^F-FDG PET study revealed that ^18^F-FDG uptake in VAT was associated with a risk of metabolic syndrome, and it reduced with adiposity by exercise [9]. Additionally, a recent ^18^F-FDG PET study reported that ^18^F-FDG uptake in VAT was positively correlated with adiponectin levels and inversely with insulin resistance, suggesting that VAT metabolism could be a proxy of VAT dysfunction [8]. VAT metabolism is expected to represent not only metabolism of the adipocyte itself, but also several complex biological processes, such as energy storage, insulin resistance, lipolysis, and adipose inflammation [8], because the VAT contains not only adipocytes, but also other cell types that contribute to its physiology and pathophysiology, including preadipocytes, mesenchymal stem cells, vascular cells, and inflammatory cells [13]. An increase in VAT mass disrupts the homeostasis of the adipose-derived molecules, such as leptin, adiponectin, apelin, and inflammatory cytokine, causing VAT dysfunction [5], and the dysregulation of these bioactive peptides could affect the brain [12]. Recently, growing evidence has suggested a critical role for VAT dysfunction in AD development [5,14,15]. However, in most previous studies, the degree of VAT dysfunction has been evaluated by measuring the bioactive peptides [3,6,16], which was not sufficient to accurately assess the degree of VAT dysfunction due to limitations in which the origin of the bioactive peptides is not clear. Interestingly, the present study showed that VAT metabolism was negatively correlated with BMI. This finding agrees with that of a previous study with cardiovascular patients that reported a negative correlation between BMI and the metabolism of the neck adipose tissue [10]. The discrepancy between BMI and VAT metabolism is consistent with the phenomenon known as the “obesity paradox”, in which a higher BMI in elderly subjects decreases the risk of AD [17]. This means that BMI is not regarded as the optimal surrogate marker for pathological obesity, as it could not differentiate between body fat and lean muscle [18]. In this context, noninvasive evaluation of VAT metabolism using ^18^F-FDG PET may be an optimal alternative for evaluating the degree of VAT dysfunction.

In the present study, there was a significant association between VAT metabolism and cerebral Aβ burden. Although ^18^F-FDG PET/CT was not used to measure VAT metabolism, several previous studies revealed the relationship between VAT dysfunction and AD pathology. A previous whole-body magnetic resonance imaging (MRI) case-control study revealed that AD patients had more volume of VAT than CU individuals, and increased leptin levels were correlated with lower CSF Aβ_1-42_ [19]. A recent clinical study reported that serum adiponectin was higher in AD patients than in MCI patients, and adiponectin CSF levels were positively correlated with Aβ_1-42_ and cognitive function, suggesting that higher serum adiponectin in AD patients constitutes a strategy to compensate for possible central signaling defects [20]. Another study with a murine model of high-fat-diet-induced VAT dysfunction reported that both adipose tissue and brain from animals fed a high-fat diet had elevated amyloid precursor protein (APP) levels localized to macrophage/adipocytes and neurons, respectively [21]. A recent animal study demonstrated that adipocyte-specific and mitochondria-targeted APP overexpressing mice had increased body mass and reduced insulin sensitivity, along with VAT dysfunction due to a dramatic hypertrophic program in adipocytes [22]. Thus, it is postulated that APP, which is expressed in both neurons and adipocytes, plays an important role in VAT dysfunction affecting AD pathology.

Although the underlying mechanism of VAT dysfunction and AD pathology is still unclear, it can be explained by dysregulation of adipokines from the VAT. VAT dysfunction causes dysregulation of adipokines, including hyperleptinemia and hypoadiponectinemia, which may contribute to AD development [4]. Leptin, which positively correlates with BMI, has been found to display neurotrophic, antiapoptotic, and neuroprotective effects [23]. Furthermore, leptin could inhibit the transport of APP by reducing beta-secretase 1 activity [24], and also facilitates the formation and motility of hippocampal dendritic filopodia, leading to enhanced synaptogenesis [25]. Thus, it is postulated that hyperleptinemia and subsequent leptin resistance are linked to AD development [22]. In addition, adiponectin, which negatively correlates with BMI, counteracts insulin resistance and exerts anti-inflammatory effects by inhibiting the expression of IL-6 or tumor necrosis factor alpha (TNFα) [26]. Since hypoadiponectinemia has been linked to several vascular risk factors, including hypertension, coronary artery disease, heart failure, cerebrovascular disease, and type 2 diabetes [27], it is presumed that hypoadiponectinemia shares a vascular risk factor and causes AD with leptin resistance [12].

Another possible mechanism to induce AD pathology by VAT dysfunction is chronic low-grade VAT inflammation, which can influence the occurrence of cerebral inflammation via circulating inflammatory mediators to increase the risk of AD development [12]. Immune dysregulation in the adipose tissues results in a chronic low-grade inflammation characterized by increased infiltration and activation of innate and adaptive immune cells, such as macrophages, dendritic cells, mast cells, neutrophils, B cells, and T cells [14]. In particular, macrophages, the predominant inflammatory cell type in VAT, are polarized into proinflammatory M1 macrophages, which secrete many proinflammatory cytokines, such as IL-6 and TNFα capable of developing chronic low-grade systemic inflammation [28]. Furthermore, VAT inflammation could induce adipocytes to produce various cytokines and chemokines, such as C-reactive protein, plasma monocyte chemoattractant protein-1, macrophage migration inhibitory factor, plasminogen activator inhibitor-1, and retinol-binding protein-4 [29]. These proinflammatory signals from the VAT may penetrate the blood–brain barrier [30] and exacerbate AD neuropathology, increasing the activity of various tau protein kinases and promoting cerebral Aβ accumulation [4,31]. A recent in vivo study with an obesity mouse model showed that a high-fat diet was associated with activation of inflammatory, endoplasmic reticulum stress, and apoptotic signals in the hippocampus [32].

The present study had some limitations. First, we used ^18^F-FDG uptake in VAT as a surrogate marker of VAT metabolism. Although ^18^F-FDG PET/CT is a well-known method for evaluating the functional activity of several organs [9,10], histopathologic studies have still not proven that ^18^F-FDG uptake is consistent with the degree of VAT dysfunction. Second, the present study could not reveal the sequential AD pathological changes according to the degree of VAT metabolism due to its cross-sectional study design. In addition, the mechanism by which VAT metabolism affects amyloid burden was not elucidated, as we did not measure serum level of adipokines or inflammatory cytokines in the present study. It is possible that the circulating plasma Aβ caused by AD can induce VAT metabolism in a vicious cycle. However, the present study provides evidence for the role of VAT in the development of AD pathology by showing the relationship between VAT metabolism and cerebral Aβ burden.

## 4. Materials and Methods

### 4.1. Study Population

A consecutive series of patients who visited our memory clinic for evaluation of cognitive function between June 2015 and January 2017 were included prospectively in this cross-sectional study. The inclusion criteria were as follows: (1) male or female aged 50–90 years; (2) patients who underwent volumetric 3-T brain MRI, torso ^18^F-FDG PET, and brain ^18^F-FBB PET within 4 weeks of their visit to the clinic; (3) patients who underwent neuropsychological evaluation, including the MMSE and the K-BNT; (4) patients for whom clinical information, including age, sex, BMI, educational level, diabetes, hypertension, hyperlipidemia, and history of cardiovascular disease, was available. The exclusion criteria were as follows: (1) patients with a MMSE score of <10; (2) patients with conditions that could affect cognition, such as vascular dementia, a history of psychiatric episodes or substance abuse, or a previous diagnosis of dementia; (3) patients who were clinically suspected of having acute infection or inflammation, or who had related findings on computed tomography (CT) or PET images. All patients were divided into three syndromal categories: CU, MCI, and dementia, based on the 2018 National Institute on Aging–Alzheimer’s Association Research Framework [33]. The study was conducted in accordance with the Declaration of Helsinki, and approved by the Institutional Review Board of Dongsan Hospital (2018-02-011). Written informed consent was obtained from all participants or their caregivers.

### 4.2. Brain MRI

In each patient, brain MRI was performed with a 3-T Signa Excite scanner (GE Healthcare, Milwaukee, WI, USA), with an eight-channel high-resolution brain coil. We obtained an anatomic image series using a three-dimensional spoiled gradient-echo sequence. Fast spin echo T2-weighted images were acquired under the following conditions: repetition time, 4000 ms; echo time, 110 ms; field of view, 210 mm; matrix, 512 × 320; slice thickness, 5 mm; and space thickness, 2 mm. The WMH volume was calculated using SPM12 (Wellcome Department of Imaging Neuroscience, Institute of Neurology, University College London, London, UK) unified segmentation routines on T1 MR images, as previously described [34]. Binary white matter masks were created from white matter segmentation maps, and WMH was segmented semiautomatically. The segmentations (blinded for clinical data) were visually checked for artifacts and segmentation errors. WMH volumes were calculated in milliliters and were normalized to the intracranial volume.

### 4.3. ^18^F-FDG PET

A PET/CT system (Biograph mCT-64, Siemens Healthcare, Knoxville, TN, USA) was used to acquire torso ^18^F-FDG PET images. To maintain a blood glucose level <150 mg/dL, all subjects fasted for at least 6 h before ^18^F-FDG PET imaging. Torso ^18^F-FDG-PET images were acquired at 50–60 min after the intravenous injection of 4.0 MBq/kg of ^18^F-FDG, in a three-dimensional mode. Nonenhanced low-dose CT was performed for attenuation correction and localization using the spiral mode at 120 kVp and 150 mAs with the True X algorithm. PET images were subjected to iterative reconstruction using ordered subset expectation maximization. Attenuation correction of the PET images was performed using attenuation data from the CT images.

To determine the degree of VAT metabolism, ^18^F-FDG uptake in VAT was measured using a dedicated PET workstation (Advantage Workstation 4.3) on torso ^18^F-FDG PET/CT images, as previously described [9]. VAT was defined as the intra-abdominal adipose tissue, and was identified in CT images based on predefined Hounsfield units (ranging from −70 to −110 HUs). The ^18^F-FDG uptake in the VAT was quantified by drawing a region of interest (ROI) around each VAT on a CT slice, which led to the consistent generation of the same ROIs on the transaxial PET images. The ROIs were drawn on each slice of three VAT areas in the right colic, left colic, and sigmoid mesenteries. The SUV was calculated as follows: SUV = tracer activity in the ROI (MBq/mL)/injected dose (MBq)/total body weight (g). The SUV_max_ was defined as the highest SUV within the ROI, and the VAT SUV_max_ was defined as the average of the SUV_max_ in the three VAT areas. The SUV_mean_ was defined as the average SUV of voxels within the VOI exceeding 42% of the SUV_max_, and the VAT SUV_mean_ was defined as the average of the SUV_mean_ for the three VAT areas. The VAT metabolism status was divided by the mean value of VAT SUV_max_ measured in healthy controls in a previous study [9]: subjects with VAT SUV_max_ < 0.74 were classified as the low VAT metabolism group, while subjects with VAT SUV_max_ ≥ 0.74 were classified as the high VAT metabolism group.

### 4.4. ^18^F-FBB PET

A PET/CT system (Biograph mCT-64, Siemens Healthcare, Knoxville, TN, USA) was used to acquire brain ^18^F-FBB PET images. Brain ^18^F-FBB PET images were acquired at 90–100 min after intravenous injection of 300 MBq of ^18^F-FBB. Quantitative analysis of the cerebral Aβ burden was conducted for the VOIs using the software program PMOD (PMOD Technologies Ltd., Zurich, Switzerland), as previously described [35]. Image processing was performed using SPM12 (Wellcome Department of Imaging Neuroscience, Institute of Neurology, University College London). Each MRI and PET image was coregistered with a standard mutual information algorithm and was spatially normalized. An automated anatomical labeling template was subsequently applied for standardized, regional brain VOI sampling of the count densities [36]. The VOIs were individually defined in the lateral frontal, lateral temporal, and lateral parietal cortices; cingulate cortex; and cerebellar cortex on ^18^F-FBB PET images. Standardized ^18^F-FBB uptake values were obtained from the defined regional VOIs. Regional SUVR_FBB_ was calculated by dividing the mean of the standardized ^18^F-FBB uptake values for the regional VOIs by that for the cerebellar cortex, as a reference region. Composite SUVR_FBB_ was calculated by averaging the SUVR of the lateral frontal, lateral temporal, and lateral parietal cortices, as well as the cingulate [37]. Subjects with a composite SUVR_FBB_ ≥ 1.39, which has previously been reported as a cut-off value that reflects an abnormally high cerebral Aβ burden, were considered positive for Aβ [38]. Patients with a composite SUVR_FBB_ < 1.39 were considered negative for Aβ.

### 4.5. Voxel-Based Analysis

A voxel-based group analysis was conducted using SPM12 (Welcome Trust Center for Neuroimaging, London, UK), implemented in MATLAB (R2018a, The MathWorks Inc., Natick, MA, USA). Each MRI and PET image was coregistered with a standard mutual information algorithm and was spatially normalized. The images were then smoothed by means of an isotropic Gaussian filter (8 mm full width at half-maximum). The ^18^F-FBB PET images were normalized to the reference region in the cerebellum. Voxel-wise-t statistics for between-group comparisons were computed, with *p*-values uncorrected for multiple comparisons. We investigated the brain areas that showed significantly increased ^18^F-FBB uptake at a peak threshold of *p* = 0.001 (uncorrected) and an extent threshold of 100 voxels. For the visualization of the t score statistics (SPM t-map), the significant voxels were projected onto a three-dimensional rendered brain or a standard high-resolution MRI template provided by SPM12, thus allowing anatomical identification. The Montreal Neurological Institute (MNI) coordinates of the local maximum of each cluster were converted into Talairach coordinates [39].

### 4.6. Statistical Analysis

All statistical analyses were performed using SPSS for Windows, version 25.0 (IBM Corp., Armonk, NY, USA). Numerical data (age, BMI, education, WMH volume, VAT SUV_max_, VAT SUV_mean_, SUVR_FBB_, and MMSE and K-BNT scores) are expressed as means ± standard deviations, and were compared among the CU, MCI, and dementia groups using one-way ANOVA. Bonferroni post hoc analysis was used for between-group comparisons. In addition, the numerical data were compared between the low and high VAT metabolism groups, and between cerebral Aβ-negative and -positive groups using two-sample *t*-tests. The *p*-values were corrected for multiple comparisons via false discovery rate correction. Fisher’s exact tests were performed to evaluate differences in the frequency of female sex, type 2 diabetes mellitus, hypertension, hyperlipidemia, and cardiovascular disease among the CU, MCI, and dementia groups, and between the low and high VAT metabolism groups. Pearson’s correlation analysis was performed to evaluate associations of the VAT SUV_max_, VAT SUV_mean_, and other variables (age, BMI, educational level, and WMH volume) with the SUVR_FBB_. Based on the univariate analysis results, variables with *p*-values < 0.05 were included in further regression analysis, in addition to age and sex. We performed multivariate linear regressions with the VAT SUV_max_ or VAT SUV_mean_ as the independent variable and the SUVR_FBB_ as the dependent variable. A *p*-value < 0.05 was considered statistically significant.

## 5. Conclusions

In conclusion, VAT metabolism was associated with AD pathology in older subjects. Our findings suggest that VAT dysfunction could contribute to the development and progression of AD. Further longitudinal studies with larger sample sizes and histopathological confirmation are necessary to evaluate the contribution of VAT dysfunction to AD development.

## Figures and Tables

**Table 1:** Characteristics of the study population.

Variables	Total (*n* = 54)	CU (*n* = 18)	MCI (*n* = 14)	Dementia (*n* = 22)	*p*
Age, years (SD)	66.4 (8.4)	62.7 (5.6)	63.9 (8.9)	71.1 (8.2)	0.002 ^1^
Sex, female, *n* (%)	34 (63.0)	11 (61.1)	7 (50.0)	16 (72.7)	0.380
Body mass index (SD)	23.3 (3.4)	24.3 (4.4)	22.6 (1.0)	22.9 (3.5)	0.277
Education, years (SD)	11.5 (6.1)	14.3 (3.5)	13.8 (6.4)	7.6 (5.6)	<0.001 ^2^
Diabetes, *n* (%)	8 (14.8)	1 (5.6%)	2 (14.3)	5 (22.7)	0.314
Hypertension, *n* (%)	16 (29.6)	1 (7.1)	4 (28.6)	11 (50.0)	0.026^1^
Cardiovascular disease, *n (%)*	6 (11.1)	1 (7.1)	2 (15.4)	3 (14.3)	0.768
Hyperlipidemia, *n* (%)	9 (16.7)	1 (8.3)	3 (21.4)	5 (22.7)	0.563
WMH volume (SD)	3.4 (5.1)	1.1 (2.2)	1.6 (2.0)	6.4 (6.5)	0.001 ^1^
MMSE (SD)	24.6 (5.3)	28.9 (1.2)	25.9 (2.9)	20.3 (5.3)	<0.001 ^2^
K-BNT (SD)	42.4 (13.5)	52.0 (4.0)	47.8 (10.1)	31.1 (12.2)	<0.001 ^2^
Aβ positivity, *n* (%)	26 (48.1)	5 (27.8)	7 (50.0)	14 (63.6)	0.077
Composite SUVR_FBB_	1.47 (0.28)	1.33 (0.11)	1.48 (0.22)	1.57 (0.36)	0.017 ^1^
VAT SUV_max_ (SD)	0.71 (0.16)	0.69 (0.17)	0.67 (0.11)	0.76 (0.17)	0.200
VAT SUV_mean_ (SD)	0.44 (0.11)	0.41 (0.11)	0.41 (0.08)	0.48 (0.12)	0.067

^1^ CU and MCI groups < dementia group in post hoc analysis; ^2^ CU and MCI groups > dementia group in post hoc analysis. CU, cognitively unimpaired; MCI, mild cognitive impairment; SD, standard deviation; WMH, white matter hyperintensity; MMSE, Mini-Mental State Examination; K-BNT, Korean-Boston Naming Test; VAT, visceral adipose tissue; SUV_max_, the maximum standardized uptake value.

**Table 2:** Comparison of clinical variables between low and high VAT metabolism groups.

Variables	Total (*n* = 54)	Low VAT Metabolism Group (*n* = 31)	High VAT Metabolism Group (*n* = 23)	*p*
Age, years (SD)	66.4 (8.4)	65.3 (8.3)	67.9 (8.5)	0.269
Sex, female, *n* (%)	34 (63.0)	18 (58.1)	16 (69.6)	0.412
Body mass index (SD)	23.3 (3.4)	23.8 (3.0)	22.5 (3.9)	0.133
Education, years (SD)	11.5 (6.1)	12.1 (5.9)	10.7 (6.3)	0.403
Diabetes, *n* (%)	8 (14.8)	4 (12.9)	4 (17.4)	0.711
Hypertension, *n* (%)	16 (29.6)	10 (37.0)	6 (26.1)	0.546
Cardiovascular disease, *n (%)*	6 (11.1)	4 (14.8)	2 (9.5)	0.683
Hyperlipidemia, *n* (%)	9 (16.7)	6 (23.1)	3 (13.6)	0.478
WMH volume (SD)	3.4 (5.08)	2.7 (4.0)	4.3 (6.2)	0.245
Cognitive stage				
CU, *n* (%)	18 (33.3)	13 (41.9)	5 (21.7)	0.234
MCI, *n* (%)	14 (26.0)	8 (25.8)	6 (26.1)	
Dementia, *n* (%)	22 (40.7)	10 (32.3)	12 (52.2)	
MMSE (SD)	24.6 (5.3)	25.8 (3.8)	23.0 (6.6)	0.245
K-BNT (SD)	42.4 (13.5)	45.0 (10.8)	38.9 (15.9)	0.107
Aβ positivity, *n* (%)	26 (48.1%)	8 (25.8)	18 (78.3)	<0.001
VAT SUV_max_ (SD)	0.71 (0.16)	0.61 (0.09)	0.85 (0.12)	<0.001
VAT SUV_mean_ (SD)	0.44 (0.11)	0.37 (0.06)	0.54 (0.83)	<0.001

SD, standard deviation; CU, cognitively unimpaired; MCI, mild cognitive impairment; WMH, white matter hyperintensity; MMSE, Mini-Mental State Examination; K-BNT, Korean-Boston Naming Test; VAT, visceral adipose tissue; SUV_max_, the maximum standardized uptake value.

**Table 3:** Regions of significantly increased cerebral Aβ burden in high VAT metabolism group compared to low VAT metabolism group in SPM analysis (*p* < 0.005 uncorrected, *k* = 100).

Regions	Brodmann Area	Size	MNI Coordinates			T Value	*p*
			X	Y	Z		
Right occipital lobe, lingual gyrus	BA 18	5896	2	−84	−8	3.56	<0.001
Right parietal lobe, precuneus	BA 19		26	−80	42	3.52	<0.001
Right parietal lobe, precuneus	BA 31		8	−68	24	3.23	0.001
Right frontal lobe, precentral gyrus	BA 44	1315	62	8	4	3.55	<0.001
Right temporal lobe, middle temporal gyrus	BA 21		64	0	−8	3.35	<0.001
Right insula	BA 13		36	10	4	3.31	<0.001
Left parietal lobe, precuneus	BA 7	4022	−18	−78	48	3.54	<0.001
Left temporal lobe, inferior temporal gyrus	BA 20		−62	−28	−16	3.44	<0.001
Left occipital lobe, inferior occipital gyrus	BA 18		−34	−90	−14	3.2	0.001
Left frontal lobe, rectal gyrus	BA 11	2258	−8	10	−24	3.37	<0.001
Right frontal lobe, inferior frontal gyrus	BA 47		26	12	−22	3.28	<0.001
Left frontal lobe, medial frontal gyrus	BA 25		−6	6	−16	3.26	<0.001
Left frontal lobe, superior frontal gyrus	BA 6	348	−2	4	54	3.3	<0.001
Left frontal lobe, medial frontal gyrus	BA 6		−4	−8	58	3.02	0.002
Left frontal lobe, inferior frontal gyrus	BA 44	450	−60	8	18	3.17	0.001
Left cerebrum, frontal lobe, precentral gyrus	BA 6		−60	6	30	3.17	0.001
Left frontal lobe, inferior frontal gyrus	BA 45		−56	20	12	3.07	0.002

VAT, visceral adipose tissue; BA, Brodmann area.

**Table 4:** Association between visceral adipose tissue SUV_max_ and cerebral Aβ burden in the overall cohort.

Regions	Univariable Model		Multivariable Model		
	*r*	*p*	Adjusted R^2^	Standardized *β* ^2^	*p*
Composite ^1^	0.414	0.002	0.195	0.359	0.007
Left lateral frontal cortex	0.360	0.008	0.113	0.360	0.008
Right lateral frontal cortex	0.353	0.009	0.140	0.302	0.025
Left lateral temporal cortex	0.379	0.005	0.127	0.379	0.005
Right lateral temporal cortex	0.338	0.012	0.147	0.278	0.038
Left lateral parietal cortex	0.428	0.001	0.218	0.369	0.005
Right lateral parietal cortex	0.421	0.002	0.201	0.366	0.005
Left cingulate	0.410	0.002	0.199	0.352	0.008
Right cingulate	0.422	0.001	0.162	0.422	0.001

^1^ Composite regional standardized ^18^F-FBB uptake value ratio (SUVR_FBB_) was calculated as the average of the SUVR of the lateral frontal, lateral temporal, and lateral parietal cortices, as well as the cingulate cortex. ^2^ Values represent the standardized linear regression coefficients (*β*) of the correlation between the visceral adipose tissue’s maximum standardized uptake value (SUV_max_) and SUVR_FBB_, after adjusting for age, sex, and white matter hyperintensity volume.

**Table 5:** Association between visceral adipose tissue SUV_mean_ and cerebral Aβ burden in the overall cohort.

Regions	Univariable Model		Multivariable Model		
	*r*	*p*	Adjusted R^2^	Standardized *β* ^2^	*p*
Composite ^1^	0.367	0.006	0.150	0.295	0.032
Left lateral frontal cortex	0.319	0.019	0.085	0.319	0.019
Right lateral frontal cortex	0.318	0.019	0.084	0.318	0.019
Left lateral temporal cortex	0.340	0.012	0.098	0.340	0.012
Right lateral temporal cortex	0.304	0.025	0.118	0.224	0.106
Left lateral parietal cortex	0.380	0.005	0.169	0.302	0.026
Right lateral parietal cortex	0.379	0.005	0.158	0.308	0.024
Left cingulate	0.360	0.008	0.152	0.282	0.039
Right cingulate	0.369	0.006	0.120	0369	0.006

^1^ Composite regional standardized ^18^F-FBB uptake value ratio (SUVR_FBB_) was calculated as the average of the SUVR of the lateral frontal, lateral temporal, and lateral parietal cortices, as well as the cingulate cortex. ^2^ Values represent the standardized linear regression coefficients (*β*) of the correlation between the visceral adipose tissue’s mean standardized uptake value (SUV_mean_) and SUVR_FBB_, after adjusting for age, sex, and white matter hyperintensity volume.

## Data Availability

All data presented in this study are available from the authors upon written request and following agreement on the intended purpose of the request. The data are not publicly available due to their containing information that could compromise the privacy of research participants.

## Supplementary Materials

The following supporting information can be downloaded at: https://www.mdpi.com/article/10.3390/metabo12030258/s1, Table S1: Comparison of cerebral Aβ burden between the low and high VAT metabolism groups; Table S2: Association between visceral adipose tissue SUV_max_ and cerebral Aβ burden in subjects with dementia; Table S3: Association between visceral adipose tissue SUV_mean_ and cerebral Aβ burden in subjects with dementia; Table S4: Association between visceral adipose tissue SUV_max_ and cerebral Aβ burden in cognitively unimpaired subjects; Table S5: Association between visceral adipose tissue SUV_mean_ and cerebral Aβ burden in cognitively unimpaired subjects; Table S6: Association between visceral adipose tissue SUV_max_ and cerebral Aβ burden in subjects with mild cognitive impairment; Table S7: Association between visceral adipose tissue SUV_mean_ and cerebral Aβ burden in subjects with mild cognitive impairment.

## References

[1] Braak H., Braak E. (1991). Neuropathological stageing of Alzheimer-related changes. Acta Neuropathol..

[2] Hardy J.A., Higgins G.A. (1992). Alzheimer’s disease: The amyloid cascade hypothesis. Science.

[3] Kiliaan A.J., Arnoldussen I.A., Gustafson D.R. (2014). Adipokines: A link between obesity and dementia?. Lancet Neurol..

[4] Misiak B., Leszek J., Kiejna A. (2012). Metabolic syndrome, mild cognitive impairment and Alzheimer’s disease—The emerging role of systemic low-grade inflammation and adiposity. Brain Res. Bull..

[5] Pichiah P.B.T., Sankarganesh D., Arunachalam S., Achiraman S. (2020). Adipose-Derived Molecules-Untouched Horizons in Alzheimer’s Disease Biology. Front. Aging Neurosci..

[6] Guo D.H., Yamamoto M., Hernandez C.M., Khodadadi H., Baban B., Stranahan A.M. (2020). Visceral adipose NLRP3 impairs cognition in obesity via IL-1R1 on CX3CR1+ cells. J. Clin. Investig..

[7] Nazeri A., Crandall J.P., Fraum T.J., Wahl R.L. (2021). Repeatability of Radiomic Features of Brown Adipose Tissue. J. Nucl. Med..

[8] Reijrink M., de Boer S.A., Antunes I.F., Spoor D.S., Heerspink H.J., Lodewijk M.E., Mastik M.F., Boellaard R., Greuter M.J., Benjamens S. (2021). [18 F] FDG Uptake in Adipose Tissue Is Not Related to Inflammation in Type 2 Diabetes Mellitus. Mol. Imaging Biol..

[9] Pahk K., Kim E.J., Lee Y.J., Kim S., Seo H.S. (2020). Characterization of glucose uptake metabolism in visceral fat by 18 F-FDG PET/CT reflects inflammatory status in metabolic syndrome. PLoS ONE.

[10] Bucerius J., Mani V., Wong S., Moncrieff C., Izquierdo-Garcia D., Machac J., Fuster V., Farkouh M.E., Rudd J.H., Fayad Z.A. (2014). Arterial and fat tissue inflammation are highly correlated: A prospective 18F-FDG PET/CT study. Eur. J. Nucl. Med. Mol. Imaging.

[11] Trayhurn P., Beattie J.H. (2001). Physiological role of adipose tissue: White adipose tissue as an endocrine and secretory organ. Proc. Nutr. Soc..

[12] Ishii M., Iadecola C. (2016). Adipocyte-derived factors in age-related dementia and their contribution to vascular and Alzheimer pathology. Biochim. Biophys. Acta BBA Mol. Basis Dis..

[13] Kahn C.R., Wang G., Lee K.Y. (2019). Altered adipose tissue and adipocyte function in the pathogenesis of metabolic syndrome. J. Clin. Investig..

[14] Zatterale F., Longo M., Naderi J., Raciti G.A., Desiderio A., Miele C., Beguinot F. (2019). Chronic Adipose Tissue Inflammation Linking Obesity to Insulin Resistance and Type 2 Diabetes. Front. Physiol..

[15] Naderali E.K., Ratcliffe S.H., Dale M.C. (2009). Obesity and Alzheimer’s disease: A link between body weight and cognitive function in old age. Am. J. Alzheimers Dis. Other Dement..

[16] Tziomalos K., Dimitroula H.V., Katsiki N., Savopoulos C., Hatzitolios A.I. (2010). Effects of lifestyle measures, antiobesity agents, and bariatric surgery on serological markers of inflammation in obese patients. Mediat. Inflamm..

[17] Sun Z., Wang Z.-T., Sun F.-R., Shen X.-N., Xu W., Ma Y.-H., Dong Q., Tan L., Yu J.-T., Alzheimer’s Disease Neuroimaging Initiative (2020). Late-life obesity is a protective factor for prodromal Alzheimer’s disease: A longitudinal study. Aging.

[18] Yang F., Wang G., Wang Z., Sun M., Cao M., Zhu Z., Fu Q., Mao J., Shi Y., Yang T. (2014). Visceral adiposity index may be a surrogate marker for the assessment of the effects of obesity on arterial stiffness. PLoS ONE.

[19] Diehl-Wiesenecker E., von Armin C.A., Dupuis L., Muller H.P., Ludolph A.C., Kassubek J. (2015). Adipose Tissue Distribution in Patients with Alzheimer’s Disease: A Whole Body MRI Case-Control Study. J. Alzheimers Dis..

[20] Letra L., Matafome P., Rodrigues T., Duro D., Lemos R., Baldeiras I., Patrício M., Castelo-Branco M., Caetano G., Seiça R. (2019). Association between adipokines and biomarkers of Alzheimer’s disease: A cross-sectional study. J. Alzheimers Dis..

[21] Puig K.L., Floden A.M., Adhikari R., Golovko M.Y., Combs C.K. (2012). Amyloid precursor protein and proinflammatory changes are regulated in brain and adipose tissue in a murine model of high fat diet-induced obesity. PLoS ONE.

[22] An Y.A., Crewe C., Asterholm I.W., Sun K., Chen S., Zhang F., Shao M., Funcke J.B., Zhang Z., Straub L. (2019). Dysregulation of Amyloid Precursor Protein Impairs Adipose Tissue Mitochondrial Function and Promotes Obesity. Nat. Metab..

[23] Guo Z., Jiang H., Xu X., Duan W., Mattson M.P. (2008). Leptin-mediated cell survival signaling in hippocampal neurons mediated by JAK STAT3 and mitochondrial stabilization. J. Biol. Chem..

[24] Fewlass D.C., Noboa K., Pi-Sunyer F.X., Johnston J.M., Yan S.D., Tezapsidis N. (2004). Obesity-related leptin regulates Alzheimer’s Abeta. FASEB J..

[25] Paz-Filho G., Wong M.L., Licinio J. (2010). The procognitive effects of leptin in the brain and their clinical implications. Int. J. Clin. Pract..

[26] Mangge H., Almer G., Haj-Yahya S., Grandits N., Gasser R., Pilz S., Moller R., Horejsi R. (2009). Nuchal thickness of subcutaneous adipose tissue is tightly associated with an increased LMW/total adiponectin ratio in obese juveniles. Atherosclerosis.

[27] Mangge H., Almer G., Truschnig-Wilders M., Schmidt A., Gasser R., Fuchs D. (2010). Inflammation, adiponectin, obesity and cardiovascular risk. Curr. Med. Chem..

[28] Haase J., Weyer U., Immig K., Klöting N., Blüher M., Eilers J., Bechmann I., Gericke M. (2014). Local proliferation of macrophages in adipose tissue during obesity-induced inflammation. Diabetologia.

[29] Monteiro R., Azevedo I. (2010). Chronic inflammation in obesity and the metabolic syndrome. Mediat. Inflamm..

[30] Banks W.A. (2005). Blood-brain barrier transport of cytokines: A mechanism for neuropathology. Curr. Pharm. Des..

[31] Kitazawa M., Cheng D., Tsukamoto M.R., Koike M.A., Wes P.D., Vasilevko V., Cribbs D.H., LaFerla F.M. (2011). Blocking IL-1 signaling rescues cognition, attenuates tau pathology, and restores neuronal β-catenin pathway function in an Alzheimer’s disease model. J. Immunol..

[32] Nakandakari S.C.B.R., Munoz V.R., Kuga G.K., Gaspar R.C., Sant’Ana M.R., Pavan I.C.B., da Silva L.G.S., Morelli A.P., Simabuco F.M., da Silva A.S.R. (2019). Short-term high-fat diet modulates several inflammatory, ER stress, and apoptosis markers in the hippocampus of young mice. Brain Behav. Immun..

[33] Jack C.R., Bennett D.A., Blennow K., Carrillo M.C., Dunn B., Haeberlein S.B., Holtzman D.M., Jagust W., Jessen F., Karlawish J. (2018). NIA-AA Research Framework: Toward a biological definition of Alzheimer’s disease. Alzheimers Dement..

[34] Van Leijsen E.M.C., Bergkamp M.I., van Uden I.W.M., Ghafoorian M., van der Holst H.M., Norris D.G., Platel B., Tuladhar A.M., de Leeuw F.E. (2018). Progression of White Matter Hyperintensities Preceded by Heterogeneous Decline of Microstructural Integrity. Stroke.

[35] No H.J., Yi H.A., Won K.S., Chang H.W., Kim H.W. (2019). Association between white matter lesions and the cerebral glucose metabolism in patients with cognitive impairment. Rev. Esp. Med. Nucl. Imagen Mol..

[36] Tzourio-Mazoyer N., Landeau B., Papathanassiou D., Crivello F., Etard O., Delcroix N., Mazoyer B., Joliot M. (2002). Automated anatomical labeling of activations in SPM using a macroscopic anatomical parcellation of the MNI MRI single-subject brain. Neuroimage.

[37] Bullich S., Seibyl J., Catafau A.M., Jovalekic A., Koglin N., Barthel H., Sabri O., De Santi S. (2017). Optimized classification of (18)F-Florbetaben PET scans as positive and negative using an SUVR quantitative approach and comparison to visual assessment. Neuroimage Clin..

[38] Barthel H., Gertz H.J., Dresel S., Peters O., Bartenstein P., Buerger K., Hiemeyer F., Wittemer-Rump S.M., Seibyl J., Reininger C. (2011). Cerebral amyloid-beta PET with florbetaben (18F) in patients with Alzheimer’s disease and healthy controls: A multicentre phase 2 diagnostic study. Lancet Neurol..

[39] Talairach J., Tournoux P. (1988). Co-Planar Stereotaxic Atlas of the Human Brain: Three-Dimensional Proportional System.

