# Supplementary material for: Association between Visceral Adipose Tissue Metabolism and Alzheimer’s Disease Pathology
Source: Metabolites. 2022 Mar 17;12(3):258. doi: 10.3390/metabo12030258 (PMC8949138; doi:10.3390/metabo12030258)
Supplement: Supplementary file 1 [file metabolites-12-00258-s001.zip › metabolites-1607784-supplementary.pdf]

**Supplementary Table S1.** Comparison of cerebral Aβ burden between the low and high VAT metabolism groups

| Variables | Low VAT metabolism group | High VAT metabolism group | *p* |
|---|---|---|---|
| Composite $SUVR_{FBB}$ [a] | 1.35 (0.16) | 1.62 (0.33) | <0.001 |
| Left lateral frontal cortex | 1.21 (0.17) | 1.46 (0.33) | 0.001 |
| Right lateral frontal cortex | 1.20 (0.16) | 1.44 (0.31) | 0.002 |
| Left lateral temporal cortex | 1.32 (0.16) | 1.56 (0.29) | <0.001 |
| Right lateral temporal cortex | 1.24 (0.15) | 1.48 (0.29) | <0.001 |
| Left lateral parietal cortex | 1.28 (0.19) | 1.59 (0.40) | <0.001 |
| Right lateral parietal cortex | 1.21 (0.19) | 1.49 (0.36) | 0.001 |
| Left cingulate | 1.48 (0.20) | 1.79 (0.40) | <0.001 |
| Right cingulate | 1.57 (0.16) | 1.85 (0.32) | <0.001 |

[a] Composite regional standardized $^{18}$F-FBB uptake value ratio ($SUVR_{FBB}$) was calculated as the average of the SUVR of the lateral frontal, lateral temporal, and lateral parietal cortices; and cingulate cortex.

**Supplementary Table S2.** Association between visceral adipose tissue $SUV_{max}$ and cerebral Aβ burden in subjects with dementia

| Regions | Univariable model | | Multivariable model | | |
|---|---|---|---|---|---|
| | *r* | *p* | Adjusted $R^2$ | Standardized $\beta$[b] | *p* |
| Composite[a] | 0.533 | 0.011 | 0.248 | 0.533 | 0.011 |
| Left lateral frontal cortex | 0.481 | 0.024 | 0.193 | 0.481 | 0.024 |
| Right lateral frontal cortex | 0.463 | 0.030 | 0.175 | 0.463 | 0.030 |
| Left lateral temporal cortex | 0.506 | 0.016 | 0.219 | 0.506 | 0.016 |
| Right lateral temporal cortex | 0.446 | 0.038 | 0.195 | 0.446 | 0.038 |
| Left lateral parietal cortex | 0.574 | 0.005 | 0.296 | 0.574 | 0.005 |
| Right lateral parietal cortex | 0.590 | 0.004 | 0.316 | 0.590 | 0.004 |
| Left cingulate | 0.509 | 0.015 | 0.336 | 0.529 | 0.008 |
| Right cingulate | 0.520 | 0.013 | 0.350 | 0.539 | 0.006 |

[a] Composite regional standardized $^{18}$F-FBB uptake value ratio ($SUVR_{FBB}$) was calculated as the average of the SUVR of the lateral frontal, lateral temporal, and lateral parietal cortices, as well as the cingulate.
[b] Values represent the standardized linear regression coefficients (β) of the correlation between the visceral adipose tissue maximum standardized uptake value ($SUV_{max}$) and $SUVR_{FBB}$, after adjusting for age, sex and white matter hyperintensity volume.

**Supplementary Table S3.** Association between visceral adipose tissue $SUV_{mean}$ and cerebral Aβ burden in subjects with dementia

| Regions | Univariable model | | Multivariable model | | |
|---|---|---|---|---|---|
| | *r* | *p* | Adjusted $R^2$ | Standardized $\beta$[b] | *p* |
| Composite[a] | 0.417 | 0.054 | 0.133 | 0.417 | 0.054 |
| Left lateral frontal cortex | 0.389 | 0.074 | 0.109 | 0.389 | 0.074 |
| Right lateral frontal cortex | 0.382 | 0.079 | 0.103 | 0.382 | 0.079 |
| Left lateral temporal cortex | 0.390 | 0.073 | 0.109 | 0.390 | 0.073 |
| Right lateral temporal cortex | 0.359 | 0.101 | 0.129 | 0.359 | 0.101 |
| Left lateral parietal cortex | 0.445 | 0.038 | 0.157 | 0.445 | 0.038 |
| Right lateral parietal cortex | 0.481 | 0.024 | 0.193 | 0.481 | 0.024 |
| Left cingulate | 0.379 | 0.082 | 0.226 | 0.426 | 0.040 |
| Right cingulate | 0.390 | 0.073 | 0.239 | 0.437 | 0.034 |

[a] Composite regional standardized $^{18}$F-FBB uptake value ratio ($SUVR_{FBB}$) was calculated as the average of the SUVR of the lateral frontal, lateral temporal, and lateral parietal cortices, as well as the cingulate.
[b] Values represent the standardized linear regression coefficients (β) of the correlation between the visceral adipose tissue mean standardized uptake value ($SUV_{mean}$) and $SUVR_{FBB}$, after adjusting for age, sex and white matter hyperintensity volume.

**Supplementary Table S4.** Association between visceral adipose tissue $SUV_{max}$ and cerebral Aβ burden in cognitively unimpaired subjects

| Regions | Univariable model | | Multivariable model | | |
|---|---|---|---|---|---|
| | *r* | *p* | Adjusted $R^2$ | Standardized $\beta^b$ | *p* |
| Composite[a] | 0.213 | 0.396 | 0.393 | 0.319 | 0.117 |
| Left lateral frontal cortex | 0.172 | 0.494 | 0.172 | 0.138 | 0.564 |
| Right lateral frontal cortex | 0.228 | 0.362 | 0.433 | 0.338 | 0.088 |
| Left lateral temporal cortex | 0.197 | 0.434 | 0.341 | 0.298 | 0.157 |
| Right lateral temporal cortex | 0.127 | 0.616 | 0.361 | 0.223 | 0.276 |
| Left lateral parietal cortex | 0.182 | 0.471 | 0.158 | 0.259 | 0.269 |
| Right lateral parietal cortex | 0.068 | 0.789 | 0.119 | 0.096 | 0.692 |
| Left cingulate | 0.262 | 0.293 | 0.380 | 0.364 | 0.080 |
| Right cingulate | 0.263 | 0.292 | 0.350 | 0.361 | 0.089 |

[a] Composite regional standardized $^{18}$F-FBB uptake value ratio ($SUVR_{FBB}$) was calculated as the average of the SUVR of the lateral frontal, lateral temporal, and lateral parietal cortices, as well as the cingulate.
[b] Values represent the standardized linear regression coefficients (β) of the correlation between the visceral adipose tissue maximum standardized uptake value ($SUV_{max}$) and $SUVR_{FBB}$, after adjusting for age, sex and white matter hyperintensity volume.

**Supplementary Table S5.** Association between visceral adipose tissue $SUV_{mean}$ and cerebral Aβ burden in cognitively unimpaired subjects

| Regions | Univariable model | | Multivariable model | | |
|---|---|---|---|---|---|
| | *r* | *p* | Adjusted $R^2$ | Standardized $\beta^b$ | *p* |
| Composite[a] | 0.119 | 0.638 | 0.345 | 0.243 | 0.242 |
| Left lateral frontal cortex | 0.045 | 0.858 | 0.151 | 0.037 | 0.878 |
| Right lateral frontal cortex | 0.140 | 0.580 | 0.386 | 0.269 | 0.185 |
| Left lateral temporal cortex | 0.205 | 0.414 | 0.360 | 0.327 | 0.118 |
| Right lateral temporal cortex | 0.101 | 0.691 | 0.349 | 0.207 | 0.318 |
| Left lateral parietal cortex | 0.105 | 0.677 | 0.125 | 0.196 | 0.409 |
| Right lateral parietal cortex | -0.071 | 0.779 | 0.051 | -0.005 | 0.983 |
| Left cingulate | 0.162 | 0.520 | 0.321 | 0.281 | 0.187 |
| Right cingulate | 0.222 | 0.376 | 0.332 | 0.339 | 0.114 |

[a] Composite regional standardized $^{18}$F-FBB uptake value ratio ($SUVR_{FBB}$) was calculated as the average of the SUVR of the lateral frontal, lateral temporal, and lateral parietal cortices, as well as the cingulate.
[b] Values represent the standardized linear regression coefficients (β) of the correlation between the visceral adipose tissue mean standardized uptake value ($SUV_{mean}$) and $SUVR_{FBB}$, after adjusting for age, sex and white matter hyperintensity volume.

**Supplementary Table S6.** Association between visceral adipose tissue $SUV_{max}$ and cerebral Aβ burden in subjects with mild cognitive impairment

| Regions | Univariable model | | Multivariable model | | |
|---|---|---|---|---|---|
| | *r* | *p* | Adjusted $R^2$ | Standardized $\beta^b$ | *p* |
| Composite[a] | 0.145 | 0.620 | -0.128 | 0.127 | 0.677 |
| Left lateral frontal cortex | 0.006 | 0.985 | -0.303 | 0.029 | 0.932 |
| Right lateral frontal cortex | 0.056 | 0.848 | -0.346 | 0.026 | 0.941 |
| Left lateral temporal cortex | 0.080 | 0.785 | -0.314 | 0.056 | 0.871 |
| Right lateral temporal cortex | 0.065 | 0.826 | -0.331 | -0.003 | 0.994 |
| Left lateral parietal cortex | 0.054 | 0.853 | -0.161 | 0.077 | 0.805 |
| Right lateral parietal cortex | 0.095 | 0.747 | -0.214 | 0.074 | 0.818 |
| Left cingulate | 0.180 | 0.538 | -0.093 | 0.156 | 0.604 |
| Right cingulate | 0.178 | 0.543 | -0.086 | 0.219 | 0.472 |

[a] Composite regional standardized $^{18}$F-FBB uptake value ratio ($SUVR_{FBB}$) was calculated as the average of the SUVR of the lateral frontal, lateral temporal, and lateral parietal cortices, as well as the cingulate.
[b] Values represent the standardized linear regression coefficients (β) of the correlation between the visceral adipose tissue maximum standardized uptake value ($SUV_{max}$) and $SUVR_{FBB}$, after adjusting for age, sex and white matter hyperintensity volume.

**Supplementary Table S7.** Association between visceral adipose tissue $SUV_{mean}$ and cerebral Aβ burden in subjects with mild cognitive impairment

| Regions | Univariable model | | Multivariable model | | |
|---|---|---|---|---|---|
| | *r* | *p* | Adjusted $R^2$ | Standardized $\beta^b$ | *p* |
| Composite[a] | 0.131 | 0.656 | -0.128 | 0.126 | 0.677 |
| Left lateral frontal cortex | 0.033 | 0.910 | -0.297 | 0.076 | 0.824 |
| Right lateral frontal cortex | 0.038 | 0.897 | -0.346 | 0.014 | 0.968 |
| Left lateral temporal cortex | 0.066 | 0.823 | -0.307 | 0.093 | 0.785 |
| Right lateral temporal cortex | 0.017 | 0.953 | -0.331 | -0.012 | 0.972 |
| Left lateral parietal cortex | 0.084 | 0.775 | -0.151 | 0.124 | 0.697 |
| Right lateral parietal cortex | 0.108 | 0.712 | -0.217 | 0.060 | 0.855 |
| Left cingulate | 0.174 | 0.552 | -0.088 | 0.168 | 0.574 |
| Right cingulate | 0.156 | 0.594 | -0.085 | 0.225 | 0.471 |

[a] Composite regional standardized $^{18}$F-FBB uptake value ratio ($SUVR_{FBB}$) was calculated as the average of the SUVR of the lateral frontal, lateral temporal, and lateral parietal cortices, as well as the cingulate.
[b] Values represent the standardized linear regression coefficients (β) of the correlation between the visceral adipose tissue mean standardized uptake value ($SUV_{mean}$) and $SUVR_{FBB}$, after adjusting for age, sex and white matter hyperintensity volume.
